# Retraction of: The cross-talk between DDR1 and STAT3 promotes the development of hepatocellular carcinoma

**DOI:** 10.18632/aging.205988

**Published:** 2024-09-15

**Authors:** Ye Lin, Haosheng Jin, Xianqiu Wu, Zhixiang Jian, Xiongfeng Zou, Jianfeng Huang, Renguo Guan, Xiangling Wei

**Affiliations:** 1 Department of General Surgery, Guangdong Provincial People's Hospital, Guangdong Academy of Medical Sciences, Guangzhou, Guangdong Province, China; 2 State Key Laboratory of Oncology in Southern China, Collaborative Innovation Center for Cancer Medicine, Department of Experimental Research, Sun Yat-Sen University Cancer Center, Guangzhou, Guangdong Province, China

**Keywords:** hepatocellular carcinoma, DDR1, STAT3, EMT, glutamine metabolism

**This article has been retracted:** Aging has completed its investigation of this paper. Multiple concerns were raised about this paper, including internal duplications and overlap with unrelated papers from different institutions. The internal duplications include Western blot images in **Figure 6F** and **6G** and repeated use of tumor specimens in **Figures 6B** and **7B**. The description of the data presented in **Figure 1C** (number of the patients) and **Figures 6H** and **7H** (liver specimens instead of lungs) does not correspond to the provided images. In addition, external duplications occurred with articles submitted to journals between February and May 2020, before the publication date of the article of the interest. These include tumor images in **Figures 6B** and **7B** that are identical to some of the tumor images in [1], immunohistochemical staining of Ki67 in **Figure 6C** that duplicates Ki67 images in [2], and immunofluorescent images in **Figure 2I** that are similar to those in [4]. In addition, **Figure 6B** has the same distinctively-scratched ruler that was used in a number of other previously and later published papers with unrelated teams of authors [1–6].

Aging contacted the authors and their institutions, and the institutions formed an ethical committee to investigate this paper. Due to the misplacement and duplication of the Figures and the loss of some key original data, the ethical committee requested that the article be retracted. Consequently, all authors agreed that the article should be retracted.
